# Super-Selective Mesenteric Embolization Provides Effective Control of Lower GI Bleeding

**DOI:** 10.1155/2017/1074804

**Published:** 2017-01-22

**Authors:** Toan Pham, Bob Anh Tran, Kevin Ooi, Marcus Mykytowycz, Stephen McLaughlin, Matthew Croxford, Iain Skinner, Ian Faragher

**Affiliations:** ^1^Colorectal Unit, Department of Surgery, Western Health, St Albans, VIC, Australia; ^2^Department of Radiology, Western Health, St Albans, VIC, Australia

## Abstract

*Introduction*. We aimed to assess the efficacy and safety of digital subtraction angiography (DSA) and super-selective mesenteric artery embolization (SMAE) in managing lower GI bleeding (LGIB).* Method*. A retrospective case series of patients with LGIB treated with SMAE in our health service. Patients with confirmed active LGIB, on either radionuclide scintigraphy (RS) or contrast-enhanced multidetector CT angiography (CE-MDCT), were referred for DSA +/− SMAE. Data collected included patient characteristics, screening modality, bleeding territory, embolization technique, technical and clinical success, short-term to medium-term complications, 30-day mortality, and progression to surgery related to procedural failure or complications.* Results*. There were fifty-five hospital admissions with acute unstable lower gastrointestinal bleeding which were demonstrable on CE-MDCT or RS over a 31-month period. Eighteen patients proceed to embolization, with immediate success in all. Eight patients (44%) had clinical rebleeding after intervention, warranting repeated imaging. Only one case (5.6%) demonstrated radiological rebleeding and was reembolized. Complication rate was excellent: no bowel ischaemia, ischaemic stricture, progression to surgery, or 30-day mortality.* Conclusion*. SMAE is a viable, safe, and effective first-line management for localised LGIB. Our results overall compare favourably with the published experiences of other institutions. It is now accepted practice at our institution to manage localised LGIB with embolization.

## 1. Introduction

Lower gastrointestinal bleeding (LGIB) is defined as bleeding from a source distal to the ligament of Treitz and represents 20–24% of all cases of gastrointestinal bleeding [1, 2]. The incidence of LGIB ranges from 20.5 to 27 cases per 100,000 in adults in the US [2, 3] and increases markedly with age, reflecting underlying diseases such as diverticular disease, angiodysplasia, colitis, and neoplasia [2–4].

Endoscopic and angiographic interventions have reduced the need for surgery in most cases of severe LGIB. Surgery would involve either directed segmental colectomy, blind segmental colectomy, or subtotal colectomy. It is usually reserved for unstable patients who have failed conservative, endoscopic, or endovascular management. There is an increased mortality rate associated with blind segmental and subtotal colectomy, particularly in elderly patients with medical comorbidities [3].

Western Health, a multicentre institution servicing a large metropolitan area in Melbourne (Australia), has recently embraced super-selective embolization of LGIB as an important treatment modality. It can obviate the need for surgery where endoscopy would otherwise be unsuccessful. Unstable patients who thus present with severe LGIB undergo screening with either contrast-enhanced multidetector computed tomography (CE-MDCT) or radionuclide scintigraphy (RS). Following identification and localisation of active bleeding in a vascular territory, the patient is transferred to the digital subtraction angiography (DSA) suite for further investigation and intervention if feasible.

The aim of this study is to review our institution's experience and compare it to the growing body of evidence on arterial embolization of LGIB.

## 2. Methods

This study is a retrospective analysis of patients presenting with LGIB treated with super-selective embolization. A search was performed through our DSA suite logbook. Our inclusion criteria consisted of all patients with confirmed LGIB (on either RS or CE-MDCT) and referred for angiography. All patients found to have active bleeding subsequently underwent endovascular treatment. Those patients who had bleeding from sites other than the lower gastrointestinal tract were excluded. The catchment for this institution consists of the western metropolitan region of Melbourne, Victoria, Australia. There are three campuses that service this area; however, patients with LGIB requiring immediate treatment are transferred to the main campus, where interventional radiological services are concentrated.

Following patients identification, a review of their medical records was performed. Data included patient characteristics such as age, sex, and comorbidities; screening modality (RS and/or CE-MDCT); bleeding territory; embolization technique; technical success; clinical success; short-term to medium-term complications including infarction, ischaemia-related stricture, and mortality up to 30 days; and need for surgery related to procedural failure or complications.

Technical success was defined as cessation of contrast extravasation as demonstrated on DSA after deployment of embolizing agent. Clinical success was defined as normalisation of vital signs, no further need for fluid resuscitation, transfusion requirement of less than two units of packed red blood cells, and no further radiologically demonstrated LGIB and subsequent further intervention for ongoing LGIB. All cases achieved 30-day follow-up and those cases performed up to May 2010 achieved 6-month follow-up.

## 3. Results

During the period from 1 January 2008 to 25 August 2010, there were 55 hospital admissions with acute unstable lower gastrointestinal bleeding which were demonstrated on CE-MDCT or RS. All of these cases progressed onto DSA with the intention of endovascular intervention.

One case was excluded on grounds of pan-gastrointestinal bleeding in a patient who was diagnosed with hemophagocytic syndrome consisting of pancytopenia and disseminated intravascular coagulation. Microcoil embolization was employed with success at controlling a significant bleeding point in the terminal ileum. However, this patient eventually died from hepatic failure.

Out of the remaining 54 cases of LGIB investigated with DSA, 18 cases (33%) had endovascular intervention. These 18 cases had a male to female ratio of 2 : 1. The median age was 74.50 years (range: 59–92). There was no active bleeding demonstrated on DSA in the remaining 36 patients; hence, no endovascular intervention was performed. Ten patients (56%) were on anticoagulation/antiplatelet medications: six patients were on aspirin alone; three were on aspirin plus clopidogrel; and one was on warfarin. The indication for warfarin was for an in situ mechanical heart valve. Patients were prescribed clopidogrel after recent insertion of coronary artery stents, while aspirin was used for prevention of cardiovascular event. Patient demographics and comorbidities are summarised in Table 1.

All but two of cases underwent embolization within 24 hours of admission. Of the two exceptions, one was initially diagnosed with periprostatic abscess causing secondary rectal bleeding. This patient's bleeding gradually worsened to the point of requiring active intervention. The other case involved embolization of colonic bleeding from an end colostomy more than 24 hours following a Hartmann's procedure for a perforated sigmoid colon.

**Figure 1:**
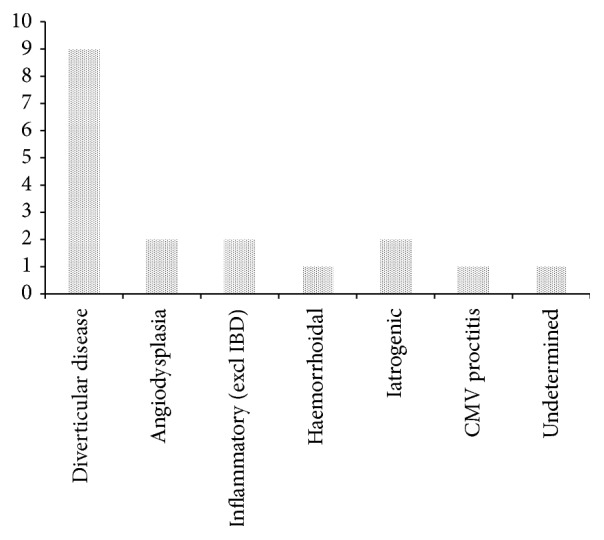
Presumptive aetiology of LGIB.

Presumptive causes of bleeding were diverticular disease (9), angiodysplasia (2), inflammatory (2), iatrogenic (2), haemorrhoidal (1), infective (1), and unknown (1) (Figure 1). Cases of note were bleeding from a rectal ulcer due to CMV proctitis and bleeding from a point in the terminal ileum of which the cause was unascertainable from the records.

**Figure 2:**
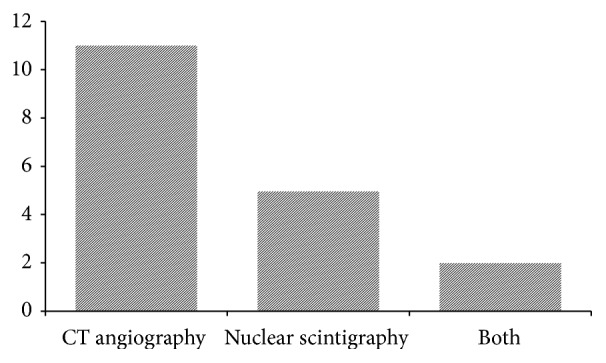
Diagnostic imaging modality performed.

CE-MDCT was performed in eleven cases, while five had radionuclide scans, and two required both modalities to identify the source of bleeding (Figure 2).

**Figure 3:**
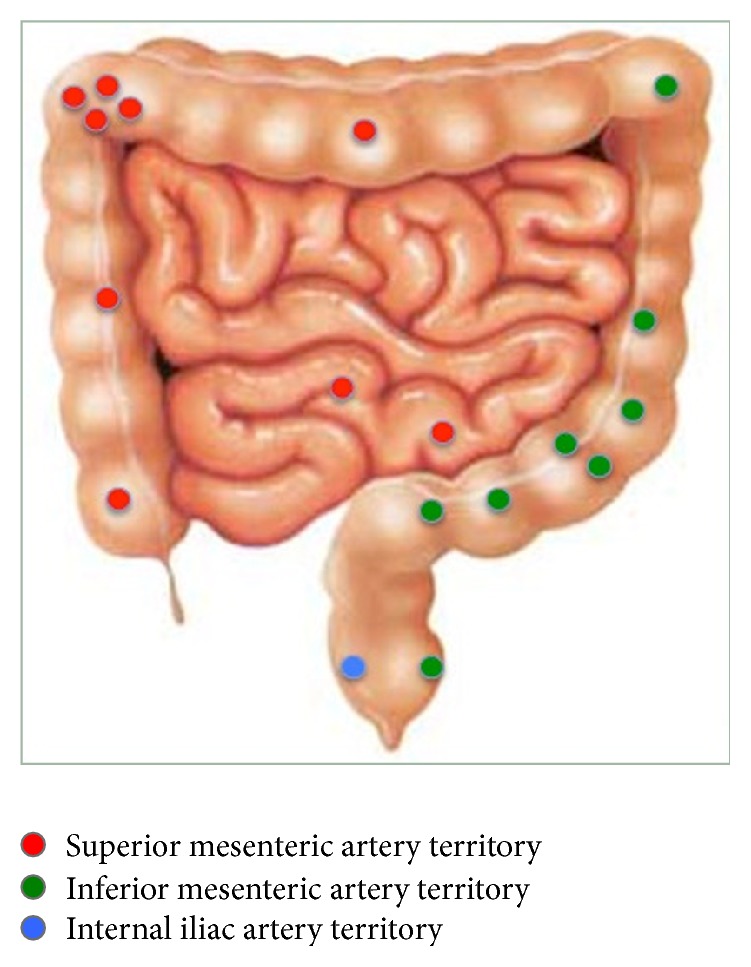
Location of bleeding sites by vascular territory.

There was an even distribution between the two major vascular territories of the lower gastrointestinal tract (Figure 3). The CMV rectal ulcer bleed was identified from the middle rectal artery (iliac artery branch) after repeated IMA studies failed to identify a bleeding point.

**Figure 4:**
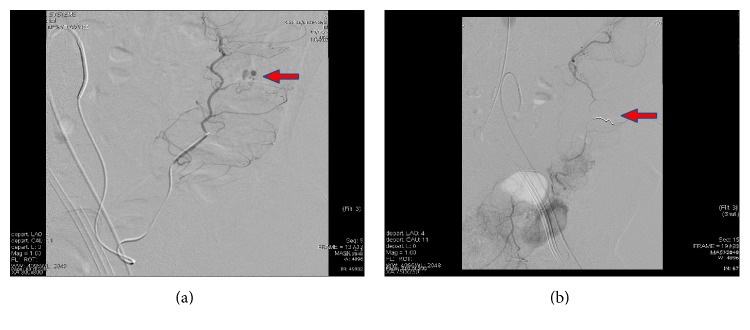
Super-selective embolization: (a) before and (b) after coil deployment.

Microcoil embolization was the preferred embolization agent and appears to be effective in achieving haemostasis (Figures 4(a) and 4(b)) with only 2 cases requiring additional Gel-Foam. Immediate haemostasis was achieved in all 18 cases after embolization. This was demonstrated by no further contrast extravasation on DSA after deployment of embolic agent. However, eight cases had clinically significant rebleeding that warranted repeated imaging within 30 days of initial intervention; three had CT angiogram, three had radionuclide scintigraphy, and two proceeded directly back to DSA. A total of 3 of the 8 patients had demonstrable rebleeding on repeat imaging but only one case required further embolization (interestingly, a case that was evaluated with DSA).

Importantly, no case of rebleeding required surgery to control bleeding. There was no 30-day mortality. There was also no documented stricture formation in the intervened segment of the gastrointestinal tract on follow-up colonoscopy, subsequent admission to hospital, or outpatient review.

## 4. Discussion

Acute LGIB is arbitrarily classified as bleeding of less than 3 days' duration and may result in haemodynamic compromise and/or the need for blood transfusion [2]. On the other hand, chronic LGIB is defined as bleeding of greater than 3 days, encompassing both occult and obscure bleeding and usually presents with iron-deficient anaemia [3]. Acute LGIB is most commonly due to diverticulosis (40%), vascular ectasia (30%), various colitis (inflammatory, ischaemic, and radiation) (20%), colonic neoplasia (14%), and anorectal causes (10%) [8]. In 80–85% of cases of LGIB, bleeding will stop spontaneously and the majority of cases will not require immediate investigation or intervention. Patients with ongoing active bleeding with or without haemodynamic compromise will need diagnostic and possibly therapeutic procedures. The current study represents a single-institution's experience in embolization for acute LGIB.

Colonoscopy has been advocated as first-line management of LGIB [2]. This has the advantage of being diagnostic with a yield of 89–97% [2] and has an accuracy rate of 72–86% [1, 3]. It also allows for the use of various haemostatic techniques where possible [3, 8]. Ideally the patient should undergo bowel preparation prior to the procedure to facilitate visualisation. However, this is not always possible especially in an unstable patient with significant haemorrhage. In this situation, the alternative is radiologic identification of a bleeding point with a view to subsequent endovascular intervention.

Three techniques are useful to localise LGIB: contrast-enhanced multidetector CT (CE-MDCT) angiography, radionuclide scintigraphy (RS), and digital subtraction angiography (DSA). Angiography has the advantage of allowing treatment during the same procedure after diagnosis has been established. It has 100% specificity but has a sensitivity of only 30–47%, requiring a relatively higher rate of bleeding of at least 1.0 ml/minute, and has a diagnostic yield ranging from 41 to 78% [3, 9]. Scintigraphy has a high sensitivity rate and can detect bleeding rates as low as 0.1 ml/minute but is less specific and unsuitable for unstable patients due to a longer study time required as well as reduced diagnostic yield with brisk bleeding [9, 10]. Recently, CE-MDCT is able to detect bleeding rates of 0.3–0.5 ml/min and is highly sensitive and specific [2, 3].

Angiographic intervention for treatment of LGIB has existed since early attempts in the 1970s. During this period, there were high rates of bowel infarction from nonselective embolization. It did not emerge as a viable alternative until the 1990s when the technology had improved. With the development of coaxial microcatheters, it became possible to carry out super-selective catheterisation of specific marginal arteries or vasa recta to deliver embolic material in the form of microcoils, Gel-Foam, or polyvinyl alcohol close to the site of bleeding. It was able to reduce the risk of infarction and decrease bleeding from collateral vessels [10]. The efficacy of super-selective embolization has been shown to vary depending on aetiology, with a greater rate of control of bleeding at 30 days in diverticular bleeding compared to nondiverticular one [10]. Following angiographic treatment, the potential complications include bowel infarction, rebleeding, and stricture formation secondary to ischaemia. However, it is understood that patients with LGIB can still pass altered blood per rectum up to 1 week after bleeding has ceased.

Three contemporary small retrospective case series from the modern era of super-selective mesenteric embolization are described in Table 2.

Waugh et al. [7] performed a review of 27 embolization cases over a period of 63 months at a metropolitan teaching hospital in Melbourne. They achieved technical success in 26 cases and clinical success in 19 cases with repeat embolization in 6 cases. Four cases had clinical symptoms of ischaemia with 2 mortalities: one due to ischaemic gut and the other related to surgical complications associated with resection of the ischaemic segment. Two cases progressed to surgery: one for ischaemia as stated above and another for ongoing LGIB despite repeated attempts of embolization. Ischaemic stricture was not an endpoint in this series.

Tan et al. [6] reviewed a series of 32 cases of mesenteric embolization over a period of 82 months at a large teaching hospital in Singapore. Technical success was achieved in 31 cases; however, clinical success was achieved only in 20 cases. 7 cases rebled with 1 managed with repeat embolization, 1 treated with colonoscopy, and 4 progressing to surgery, and the remaining case was managed conservatively due to underlying metastatic disease. Three cases underwent bowel resection at the treating surgeons' preference despite no rebleeding or ischaemia and one case had a segmental colectomy for ischaemia. It is important to note that only* 5 cases* underwent surgery for indications related to LGIB and ischaemia. There were 3 mortalities in this series; however, only one case was directly related to intervention. Ischaemic stricture was not reported.

The third series was by Rider et al. [5] from the Ochsner Clinic (New Orleans, USA) consisting of 24 cases over a 2-year period. They achieved technical success in all cases. One case rebled and was unsuccessfully reembolized, thus requiring sigmoid colectomy. Two cases developed ischaemia and underwent segmental colectomy. There was no mortality. One case developed an ischaemic stricture that required subsequent surgical intervention.

We have shown that acute unstable LGIB can be managed effectively using super-selective mesenteric embolization following radiological localisation. In comparison with the other 3 studies, our study had better outcomes when comparing the proportion of patients who had postembolization ischaemia, the proportion of patients who progressed on to surgery, 30-day mortality, and the proportion of patients who developed ischaemic stricture after embolization. However, we had a higher proportion of patients who rebled after embolization and patients who required repeat embolization compared with Rider et al.'s and Tan et al.'s studies but the rates were lower when compared with Waugh et al.'s study.

Based on these positive preliminary results, our centre has adopted a protocol of using CE-MDCT to localise the vascular territory immediately following resuscitation and stabilisation of all acute unstable LGIB. However, this study is limited by factors inherent to retrospective case series type studies as well as having a small cohort of patients. It is thus underpowered to draw any necessary statistically significant conclusions. Our results suggest that further research is warranted with prospective recruitment of cases and follow-up using a defined protocol.

## 5. Conclusion

Super-selective mesenteric embolization is a viable, safe, and effective first-line management for localised LGIB. Our results overall compare favourably with the published experiences of other institutions. It is now accepted practice at our institution to manage localised LGIB with embolization.

## Figures and Tables

**Table 1:** Demography.

Demography	Number
Gender	
(i) Male	12 (67%)
(ii) Female	6 (33%)
Median age	74.50 (range: 59–92)
Comorbidities	
(i) Ischaemic heart disease	7 (39%)
(ii) Atrial fibrillation	3 (17%)
(iii) Hypertension	12 (67%)
(iv) Diabetes	6 (33%)
Anticoagulation	
(i) Aspirin	6 (33%)
(ii) Aspirin + clopidogrel	3 (17%)
(iii) Warfarin	1 (6%)

**Table 2:** Comparisons of outcomes with other series.

Outcomes	Current study (Australia, 2011) *N* = 18	Rider et al. [5] (USA, 2009) *N* = 24	Tan et al. [6] (Singapore, 2008) *N* = 32	Waugh et al. [7] (Australia, 2004) *N* = 27
Immediate hemostasis	100%	100%	97%	96%
Rebleeding	19%	4.3%	63%	29.6%
Repeated embolization	6%	4.3%	3%	22%
Ischaemia	0%	8.7%	3%	15%
Progression to surgery	0%	12.5%	28% (4 rebleeding; 1 incomplete hemostasis; 1 ischemia; 3 surgeon decision)	7.4%
30 d mortality	0%	0%	9%	7.4%
Ischaemic stricture	0%	4.3%	Not reported	Not reported
